# Symptom severity and mindreading in narcissistic personality disorder

**DOI:** 10.1371/journal.pone.0201216

**Published:** 2018-08-15

**Authors:** Elena Bilotta, Antonino Carcione, Teresa Fera, Fabio Moroni, Giuseppe Nicolò, Roberto Pedone, Giovanni Pellecchia, Antonio Semerari, Livia Colle

**Affiliations:** 1 Third Center of Cognitive Psychotherapy, Rome, Italy; 2 Department of Psychology, University of Campania Luigi Vanvitelli, Caserta, Italy; 3 Department of Psychology, Center of Cognitive Science, University of Turin, Turin, Italy; Leibniz Institute for Educational Trajectories, GERMANY

## Abstract

**Background:**

Grandiose narcissism has been associated with poor ability to understand one’s own mental states and the mental states of others. In particular, two manifestations of Narcissistic Personality Disorder (NPD) can be explained by poor mindreading abilities: absence of symptomatic subjective distress and lack of empathy.

**Methods:**

We conducted two studies to investigate the relationships between mindreading capacity, symptomatic subjective distress and narcissistic personality. In the first study (N = 246), we compared mindreading capacities and symptomatic distress in three outpatient samples: narcissistic patients (NPD); patients with other Personality Disorders (PD); patients without PD. In the second study (N = 1357), we explored the relationships between symptomatic distress, mindreading and specific NPD criteria.

**Results:**

In the first study, the NPD patients showed poorer mindreading than the patients without PD and comparable to patients with other PDs. Symptomatic subjective distress in the narcissistic group was less severe than in the other PDs group and comparable to the group without PDs. However, no relationship emerged between mindreading and symptomatic subjective distress. In the second study, taking the clinical sample as a whole, symptomatic distress appeared negatively linked to grandiosity traits, while mindreading scores were negatively linked to empathy.

**Conclusions:**

NPD showed specific mindreading impairments. However, mindreading capacity did not appear to be directly connected with subjective distress, but did appear to be connected with specific aspects of narcissistic pathology.

## Introduction

The fifth edition of the Diagnostic and Statistical Manual of Mental Disorders (DSM-5) has inherited from previous editions the description of Narcissistic Personality Disorder (NPD) as a pattern of grandiosity, pursuit of admiration and lack of empathy [1]. Individuals with this disorder show scant consideration for the feelings of others and exhibit disagreeable behaviours and feelings of envy and contempt. Several authors have emphasized that this description of DSM-5 captures only one aspect of pathological narcissism, which corresponds to the grandiose phenotype. However, there is a body of literature which suggests the existence of different subtypes of narcissism [2–7]. Although every subtype of narcissism is characterized by emotional dysregulation and unstable self esteem instability [8–11], grandiose narcissists typically show an inflated self image and lack of depressive symptoms and subjective distress. The absence of subjective distress as well as lack of empathy have been associated with grandiose NPD patients’ impaired understanding of their own mental states and of the mental states of others [12–17]. This understanding has been variously defined in the literature as mindreading [18], mentalization [19], metacognition [20], theory of mind [21]. In this article we will use the all-inclusive term *mindreading* to refer to the overall ability, and the other terms when we refer to the terminology of the cited works. Several clinicians have associated NPD with impaired mindreading. According to Dimaggio and colleagues [10], metacognitive deficits play an important role in shaping and maintaining the pathology and the problematic interpersonal relations of patients with NPD. Mizen [22] suggested that narcissistic patients present generalized failures of symbolization and find it difficult to represent affective and visceral feelings in words. Krystal [23] found additionally that narcissistic individuals present marked difficulties in identifying and verbalizing emotions; in other words, they are alexithymic, which is associated by Nemiah and Sifneos [24] with susceptibility to psychosomatic disorders. Although it could be hypothesized that a deficit in mindreading might explain lower subjective distress and lack of empathy among NPD patients, empirical data supporting this view are still inconclusive. Research has not demonstrated conclusively that grandiose NPD have difficulties in understanding their own mental states.

Diamond and colleagues showed that patients with comorbid narcissistic and borderline personality disorder scored low on mentalizing [25]. In a set of single case studies, Dimaggio and colleagues [26] found that patients with NPD displayed difficulties in recognizing the thoughts and emotions which make up their mental states, and also in making connections between internal states and their external causes.

However, other studies on the relationship between narcissism and alexithymia have produced contradictory results and conclusions. In one research study on patients with eating disorders, Lawson and colleagues found that the presence of narcissistic traits was linked to alexithymia [27], while other studies carried out on samples of patients with Personality Disorders (PD) established no such connection between narcissism and alexithymia [28, 29]. Although some authors have suggested that grandiose narcissists may be unable to access the fragile aspects of their emotional lives [16], it remains unclear whether this difficulty might be due to a specific deficit in mindreading, or if it could be traced back to motivational factors.

There is in fact a certain amount of evidence indicating that narcissistic patients are hyper-sensitive to information that could cause them psychological distress but at the same time, on the level of conscious awareness, apparently oblivious to such information. Cascio and colleagues, for instance, found that under experimental conditions of social exclusion the narcissistic individuals showed hyperactivity of cerebral areas anatomically defined as a social pain network, but that this hyperactivation was not reflected in their subjective experience as referred in their self-reports [30]. Analogously, Horvath and Morf [31] found that in a non-clinical sample the subjects with the highest levels of narcissism demonstrated hypervigilance for information conveying worthlessness, but were also apparently unaware of this information. These results pose the question whether and to what extent this absence of subjectively perceived distress can be attributed to a motivational repression mechanism, as Horvath and Morf suggest, or whether it can instead be attributed to a failure of symbolization of affective and visceral experience at a representational level of consciousness [22]. It is also possible that both factors are involved in a circular mutually reinforcing interaction, a vicious circle which renders patients with grandiose narcissism increasingly incapable of understanding their own psychological experiences.

A second aspect of narcissistic pathology which has been seen as linked to mindreading deficits is lack of empathy [10]. Although lack of empathy represents a core aspect in narcissism, it can also be observed in other disorders [11]. In Section III of DSM-5, lack of empathy is conceived as a general factor of personality pathology. However, according to Section III, different manifestations of personality pathology might show different kinds of empathic impairments. For example, lack of empathy in Antisocial PD is described as a lack of concern for feelings, needs or suffering of others, while in Avoidant PD it is related to preoccupation with or sensitivity to criticism and rejection [1]. Such differences in empathic impairments within personality disorders can be traced back to the multifaceted nature of empathy, which includes different components that in certain clinical conditions can appear separated and disentangled. Research studies have concentrated on the ways narcissism relates to cognitive empathy on the one hand, and to emotional empathy on the other. Cognitive empathy is related to understanding other people’s mental states and to understanding the perspectives which influence the mental states experienced by others. This level of perspective taking is similar to Piaget’s concept of cognitive decentration [32] and also to allocentric perspective [33], and it refers to the capacity to apprehend the mental states of others independently of our own relationship to them, departing from what we know of the other person’s value system and preferences and values [34]. Emotional empathy refers to the emotional response evoked by perceived mental states of others and may include both a sympathetic reaction to the other person’s distress and/or feelings of personal distress [35]. In Section III of DSM-5, low empathy in narcissism is described in terms of lack of cognitive empathy and high egocentricity: "*impaired ability to recognize or identify with the feeling and the needs of others; excessively attuned to reactions of others*, *but only if perceived as relevant to self; over or underestimate of own affect on others*" (p. 890) [1]. Empirical research has yielded only mixed evidence in support to this definition. Most of studies were conducted on non-clinical populations. Results indicate a strong overall relationship between narcissism and deficient emotional empathy, and a weak connection with dysfunctional cognitive empathy [36]. In one of the few clinical studies of patients with NPD, Ritter and colleagues [37] compared the empathy profile of NPD patients with empathy profiles of borderline patients and a healthy control group, using both self-report scale and laboratory test. The results obtained show low levels of emotional empathy in narcissists but normal levels of cognitive empathy. Analogously, in a similar study of a patient sample with PDs, Hengartner and colleagues also found low levels of emotional empathy [38]. Further, in a sample of narcissistic subjects with psychopathological traits, Marcoux and colleagues highlighted that the subjects of the study presented neurosensory hyperactivity while observing pain inflicted on others, a response which was not matched with any reported subjective perceptions of distress [39]. According to the authors, these data would appear to indicate that narcissistic individuals are capable of recognizing and reacting to the suffering of other persons, but that they are motivated to disregard distress in others.

However, although such studies provide some evidence for the hypothesis that narcissistic persons may possess a normal capacity for understanding other minds, there are other considerations which suggest that caution may be warranted with regard to these findings. Marissen and colleagues [40] found that patients with NPD have difficulties with recognition of the emotions of others, an ability which is usually considered indicative of cognitive empathy. Given the interpersonal difficulties of patients with high levels of narcissism and their inability to establish satisfying relationships, it is plausible that these patients may lack the capacity to assume other people’s perspectives in everyday contexts [41].

The fact that it is difficult to demonstrate the presence of mindreading impairments in patients with NPD may depend upon a number of different confounding factors, which include the limitations of self-reports and of laboratory tasks, the complexity of mindreading abilities and the impact of the general severity of the personality pathology. Some recent data appear to validate to a certain extent the self-reports of narcissistic patients on their own pathological symptoms [42]. In another study, however [43], individuals with narcissistic traits appear to overestimate their own competence at understanding mental states. Although considerable progress has been made in devising laboratory tasks which are closer to real life situations, such as the MASC [44], their ecological validity is still questionable [45] and the gap between laboratory and real life conditions remains hard to bridge. There is a difference between understanding the perspective of an agent in a movie, for example, and understanding the perspective of a person one is directly involved with in an interpersonal exchange.

Moreover, mindreading is a complex process which calls for the use of a variety of implicit and explicit abilities, with different domains and different levels of complexity [34, 46, 47]. Staying with an example for cognitive empathy, two individuals might both easily recognize that Joe is angry. However, while one of them may be able to explain this emotion in terms of Joe’s character and what has just happened to upset him, independently of his own relationship to Joe (providing a *decentred* reading of what is going on or, to use Frith and De Vignemont’s expression, an *allocentric* interpretation), the second individual may remain within his own *egocentric* perspective and may simply conclude that Joe must be angry with him. It is possible that some studies may miss mindreading impairments because the mindreading aspects they are investigating are not those which are specifically impaired in the sample under consideration.

Another possible confounding factor may be the effect of general personality pathology as measured on the basis of the number of PD comorbidities or on the basis of numbers of criteria met [48]. For example, in a study of a large sample of patients with personality disorders, it was found that mindreading impairment was associated more with general personality severity, measured on the basis of numbers of criteria met, than with specific styles or categories [49].

For the current study, in order to minimize the effect of general personality severity, we selected only subjects who met criteria for a single PD diagnosis. In order to limit bias in self-assessments of narcissistic subjects, we used both self-report measures of mindreading and semi-structured interviews. Additionally, the interview we used is constructed around a real life experience of the subject which he or she considers emotionally meaningful.

Our aim was to explore the relationships between mindreading and narcissistic personality functioning. The study is divided into two parts. In the first part we investigated whether patients with grandiose NPD present with low subjective symptomatic distress and poor mindreading skills and whether there is a connection between these two elements. In order to do this, we selected from a large sample of participants a group of patients with NPD, and compared it with a heterogeneous group of patients with a variety of PD diagnoses and with a patient group without PD diagnoses. Consistently with other studies, we expect that the patients with grandiose NPD will show less subjective symptomatic distress than the other two groups. Moreover, on the basis of our previous research [49], we expect that the patients with PDs will show poorer mindreading ability than the patients without PD and that in the semi-structured interview the NPD patients will perform at the same mindreading level as the other PD patients and at a lower level than the patients without PD. However, taking into account that narcissists are prone to positive bias when assessing their own capacities, we expect that their self-reports will characterize their own mindreading capacities as superior to those of the other PD patients. Finally, considering the grandiose NPD patients, we expect to find a link between poor mindreading as evaluated in the semi-structured interview and low subjective symptomatic distress.

In the second part, conducted on a larger sample, we focused on narcissistic criteria rather than on narcissistic diagnosis in order to explore their relationships with symptomatic distress and mindreading. Congruently with previous research studies which have found a relationship between avoidance of psychological distress and the maintenance of grandiosity, we expect to find an inverse relationship between grandiosity trait and symptomatic distress. Given that narcissists tend to undermine their own interpersonal aims through counterproductive behaviors [41], we also expect to find an inverse relationship between mindreading ability and those NPD criteria which are likely to generate counterproductive social behaviors, specifically: lack of empathy, arrogance and sense of entitlement.

## Study 1

In the first study we mainly aimed at investigating the differences in perceived symptoms severity and mindreading among three different groups: Narcissistic PD (NPD), patients with other personality disorders than narcissistic (Other PD), and patients with no personality disorder (No PD). Secondly, we aimed at investigating the relationship between subjective perceived distress and mindreading among NPD patients. Finally, we aimed to observe discrepancies between self-reported and observed measures, especially measures of mindreading, and to compare the incidence of such discrepancies in the three groups. In order to do this, we conceived alexithymia as an indirect measure of self-report mindreading.

### Materials and methods

#### Participants

Participants were adult patients who attended an Italian outpatient clinic for consultation or treatment between 2011 and 2016. From a large sample of patients seeking treatment (N = 1346), we selected three groups for a total of 246 patients. The mean (SD) age of the selected sample was 34.90 (11.61) years (range 18–73 years). A total of 113 (46%) were male and 133 (54%) were female. The first group (N = 32; 13% of the sample) included participants who met the DSM-IV criteria for Narcissistic Personality Disorder (NPD, 5 or more criteria) and were below the cut-off values for all the other PDs. The second was Other PD group (N = 157; 63.8% of the sample) and included patients who met the criteria for only one Personality Disorder diagnosis other than Narcissistic, while Narcissistic Personality Disorder criteria were kept equal to zero. This restriction was chosen in order to reduce the potentially confounding effect deriving from the overlap among different personality disorders. The third group was made up of No PD patients (N = 57; 23.2% of the total sample) including participants who met one or less criterion for a personality disorder, according to the Diagnostic and Statistical Manual of Mental Disorders. Patients with multiple Personality Disorder diagnoses, neurological disorders, psychotic disorders, and active substance dependence were not included in the selected sample. Table 1 shows the characteristics of the selected sample. The dataset is available in the supporting information (S1 File).

**Table 1:** Demographic and diagnostic characteristics of the sample.

Group				N			Gender			Age, Mean (SD)	
No Personality Disorder				57			29 M/28 F			36.5 (13.30)	
Narcissistic Personality Disorder				32			23 M/9 F			37.06 (9.61)	
Other Personality Disorder				157			61 M/96 F			33.98 (11.28)	
**N of Diagnosis for Personality Disorder**											
AV	DEP	O-C	PA		DE	PAR		SZ	HIS		BDP
42	18	40	13		12	1		1	2		28
**N of Diagnosis for Axis I symptoms**											
**Diagnosis**				**No PD**			**Narcissistic PD**			**Other PD**	
No Axis I diagnosis				13			10			23	
Mood disorders				13			13			53	
Anxiety disorders				25			7			60	
Somatoform Disorders				6			0			9	
Other				0			2			12	

M, male; F, female; AV, avoidant PD; DEP, dependent PD; O-C, obsessive compulsive PD; PA, passive-aggressive PD; DE, depressive PD; PAR, paranoid PD; SZ, schizoid PD; HIS, histrionic PD; BDP, Borderline PD. In the Axis I diagnoses, "Other" included sexual dysfunctions, eating disorders and adjustment disorders.

#### Procedure

PD evaluation and diagnosis were administered by a clinical team of psychologists and psychiatrists from the Third Center of Cognitive Psychotherapy of Rome, Italy. All participants signed written consent forms before participating in the study. Following informed consent, all participants completed the questionnaires described below and were then interviewed in order to evaluate their metacognition levels. The protocol was approved by the Scientific and Research Ethic Committee at School of Cognitive Psychotherapy, Rome, Italy.

#### Measures

The Structured Clinical Interview for DSM-IV axis I and II. The DSM-IV axis I and II diagnoses were obtained using the Structured Clinical Interview for DSM Axis I and II Disorders (SCID-I and SCID-II) [50, 51]. Internal consistency of PDs traits ranged from 0.75 and 0.85 for most of the PD diagnoses; only four PD diagnoses, obsessive compulsive, dependent, schizotypal and passive-aggressive, had alphas above 0.65. In previous studies we observed that inter-rater reliability was adequate for both trait scores (a two-way mixed absolute agreement model for intra-class correlations coefficients ranged between 0.84 and 0.99; mean, 0.93) and categorical diagnoses (average *κ* = 0.90) [52].

The Symptom Checklist-90-R. The Symptom Checklist-90-R (SCL90-R) [53] is a 90 item self-report inventory designed to measure the current (state) psychological symptom status of clinical patients. Participants are requested to fill out a questionnaire which elicits their subjective levels of symptom related distress (90 symptoms are listed) over the two previous weeks, on a scale ranging from 1 = *not at all* to 4 = *very much*. The SCL-90-R measures nine primary symptom dimensions (i.e., somatization, obsessive-compulsive, interpersonal sensitivity, depression, anxiety, hostility, phobic anxiety, paranoid ideation, and psychoticism and generates an estimate of global psychopathology, the Global Severity Index (GSI). For the purposes of this study, we used the GSI as a measure of perceived symptomatic distress.

The TAS-20 scale. Alexithymia was used here as a measure of self-reported mindreading and was measured by the Italian version of the Toronto Alexithymia Scale (TAS-20) [54]. The TAS-20 consists of 20 items rated on a 5-point Likert scale (ranging from 1 = *strongly disagree* to 5 = *strongly agree*) and measures three facets: Difficulties Identifying Feelings; Difficulties Describing Feelings; and Externally Oriented Thinking, which refers to a specific tendency to focus on superficial matters and to avoid emotional thinking [55]. We used the scores of each dimension and the total score for the analyses (α = .71). The total score ranged from 20 to 100, with a score ≤51 classifying an individual as non-alexithymic and a score ≥61 classifying the individual as alexithymic; scores between 52 and 60 classify the individual as borderline.

The Metacognition Assessment Interview (MAI) [56, 57] is a semi-structured clinical interview constructed around a narrative task and designed to elicit and evaluate the metacognitive abilities of participants. Participants are asked to recall and give a brief account of a psychologically meaningful experience or event situated during the previous six months. The reported experience must be autobiographical and must involve at least one other person, so that interviewers can evaluate participants’ ability to understand mental states of others. Once this brief narrative task is completed, participants are asked a series of specific questions based upon the account and designed to evaluate four metacognitive subfunctions: *Monitoring*, *Integration*, *Differentiation*, *and Decentration*.

*Monitoring* refers to the ability to identify and label components of mental states: emotions, thoughts, motivations and desires. Individuals with poor monitoring ability find it difficult to describe their internal states and to explain the motivations underlying their behavior.

*Integration* refers to the ability to reflect upon different mental states and to identify internal contradictions and potential patterns. Integration promotes behavioural coherence by allowing us to organize mental contents adaptively in terms of relative importance and subjective priorities. Conversely, individuals with poor integration ability find it difficult to maintain coherence between mental processes and behaviors.

*Differentiation* refers to the ability to distinguish between internal psychological contents (i.e., mental states) and external reality. Individuals with poor differentiation ability find it difficult to establish and maintain a critical distance to their own subjective mental representations.

*Decentration* refers to the ability to assume the perspectives of others and to make plausible hypotheses about their mental states. This is a form of perspective-taking similar to the concept of decentration as described by Piaget [32] and also to allocentric perspective, in the definition of Frith and De Vignemont [33]. Individuals with poor decentration ability find it difficult to reflect on other people’s intentions, thoughts and desires from the perspective of the other rather than from their own standpoint. In the present study, MAI was administered at an early stage of the project and before engaging patients in clinical treatment. Inter-rater reliability was tested in a previous study [56].

### Data analyses

In order to test our hypothesis, a series of univariate ANOVA were performed to compare the mean scores of the three groups (No PD, NPD and Other PD) on alexithymia (TAS-20) and symptoms severity (GSI; SCL-90-R) and metacognition scores (MAI interview). Post-hoc tests with Least Significant Difference (LSD) procedure were computed to test the differences between the three groups, if the omnibus F test was significant. A bivariate correlation was performed in order to test the relationship between self-report measures of perceived psychological distress (GSI), alexithymia and mindreading. Statistical analyses of data were performed using SPSS 20.0.

### Results and discussion

To maintain the rate of type I error close to the nominal .05 level, we performed a Multivariate analysis of variance (MANOVA) considering all the dependent variables (TAS-20, SCL-90-R and MAI) before approaching the univariate tests. The Multivariate effect turned out to be significant (Wilk's λ = .58; F (34,400) = 3.67; p < .001).

Table 2 shows the mean scores of patients with no PD, NPD and patients with other PD on the following dependent variables: alexithymia (TAS-20) subscales and global score, symptoms severity (GSI) and metacognition (MAI) dimensions and global score. Concerning the TAS subscales *Difficulties in identifying feelings* and *Difficulties describing feelings*, no significant difference between the No PD group and the NPD group was found, but both groups showed lower scores than patients with Other PD.The differences among the three groups were not significant for the TAS subscale *Externally oriented thinking*. Concerning the results on symptoms severity (GSI-SCL-90-R) [53], NPD showed lower levels of perceived suffering, compared to Other PD, and were more similar to No PD.

**Table 2:** Alexithymia, symptoms severity and metacognition as a function of absence of PD, narcissism, and other PDs.

	No PD M (SD)	NPD M (SD)	Other PD M (SD)	F (df)	Effect size (Cohen's *f*)
Difficulties in identifying feelings	14.81^a^ (4.91)	15.84^a^ (5.54)	19.46^b^ (6.99)	13.02** (2, 243)	.379
Difficulties in describing feelings	12.47^a^ (2.87)	13.31^a^ (3.08)	14.52^b^ (2.91)	10.88** (2, 243)	.295
Externally oriented thinking	16.51^a^ (3.96)	15.59^a^ (4.02)	17.35^a^ (4.59)	2.47 (2, 243)	.148
TAS total	43.79^a^ (8.05)	44.75^a^ (8.79)	51.33^b^ (10.41)	15.68** (2, 243)	.379
Symptoms severity	.76^a^ (.49)	.98^a^ (.54)	1.2^b^ (.64)	15.06** (2, 243)	.329
Monitoring	14.17^a^ (2.53)	12.22^b^ (2.90)	12.63^b^ (2.94)	6.06* (2, 217)	.239
Differentiation	14.09 ^a^ (2.12)	10.85^b^ (2,48)	12.07^c^ (2.52)	17.97** (2, 217)	.380
Integration	13.96^a^ (2.62)	10.81^b^ (3.27)	11.36^b^ (2.82)	16.92** (2, 217)	.382
Decentration	13.34^a^ (2.71)	10.44^b^ (2.83)	11.65^c^ (2.87)	10.17** (2, 217)	.307
MAI total	55.55^a^ (8.45)	44.33^b^ (9.38)	47.71^b^ (9.51)	16.54** (2, 217)	.394

Means with different superscripts differ at p < .05 (LSD test).

** = p < .001

* = p < .01.

Symptoms Severity = GSI-SCL-90-R. For alexithymia and GSI No PD (N = 57); NPD (N = 32); Other PD (N = 157). For MAI No PD (N = 47); NPD (N = 27); Other PD (N = 146).

Concerning our second hypothesis, NPD showed lower scores of mindreading compared to No PD, as hypothesized. The differences between the groups on the global score of MAI were significant. The post hoc performed showed NPD had significantly lower mean scores than the No PD group, while NPD did not differ from the Other PD group. Other PD and No PD differed significantly.

Concerning the subscales of MAI, NPD patients showed the lowest scores in the *Differentiation* and *Decentration* dimensions. On the dimensions *Monitoring* and *Integration*, NPD showed lower scores than No PD, but equal to Other PD group.

No significant correlation emerged between subjective symptomatic distress and alexithymia (*r* = .23; p = .201), symptomatic distress and metacognition (*r* = .16; p = .422), and alexithymia and metacognition (*r* = -.20; p = .316) in the NPD group. The association between symptomatic distress and alexithyimia turned out significant (*r* = .41, p < .001) in the Other PD group. In the same group, the correlation between symptomatic distress and metacognition was not significant (*r* = -.07; p = .427), as well as the correlation between alexithymia and metacognition (*r* = -.02; p = .798). In the No PD group, no significant correlation emerged between subjective symptomatic distress and alexithymia (*r* = .21; p = .111), symptomatic distress and metacognition (*r* = -.07; p = .647), and alexithymia and metacognition (*r* = -.12; p = .430). In an exploratory fashion, we compared the magnitude of the correlations (relying on r-to-z transformations) among symptomatic distress, metacognition and alexithymia across the three groups (NPD, No PD and Other PD) to investigate further whether the association (or lack thereof) between mindreading and subjective distress was specific for the NPD group. Comparing the coefficients across pairs of groups failed to yield any significant difference in the magnitude of the associations across groups (all ps >.15). On the whole, this investigation on the association between subjective distress and mindreading appears to falsify the hypothesis that the relative absence of subjective distress among NPD patients is associated with low mindreading.

In this first study we set out to test the hypothesis that patients with grandiose narcissism present both low symptomatic distress and poor mindreading ability and to investigate possible connections between these two elements. As predicted by the hypothesis, patients with grandiose NPD presented with significantly lower levels of symptomatic distress than the other PD patients. Their levels of symptomatic distress are in fact comparable to those reported by patients with no PD diagnosis. This lack of differentiation between the NPD group and the no PD group was an unexpected result. Since the sample consisted of patients actively seeking treatment in an outpatient setting, we had considered it plausible that the relational difficulties created by narcissistic pathology might have developed to a point that would ultimately favour the perception of psychological distress, even if levels of distress might have remained significantly lower than in the other PD group [15].

As predicted by the hypothesis, when measured with the MAI, the mindreading performance of NPD patients was significantly worse than that of the no PD patients and similar to that of the other PD patients. The results obtained through the semi-structured interview are not consistent with the results of the self-reports measures: compared with self-assessments of other PD, NPD rate themselves as less alexithymic. These data support the idea that narcissists may be prone to bias when assessing their ability to understand their own emotional states. Analogously, Ames and Kammrath [43] found that more severe narcissism is associated with a greater discrepancy between effective and self-assessed competence in understanding mental states of others. It is interesting to note that in the MAI, on the decentering sub-function, which evaluates ability to understand the minds of others, the performance of the narcissistic patients was slightly but significantly worse than that of the patients with other PDs. This result suggests that in real life the narcissistic patients may have difficulties understanding the minds of others, at least in situations of more sophisticated perspective-taking and when they are personally involved in the relationship.

On the whole, these data show that patients with grandiose narcissism are overconfident when assessing their own mental states and those of others but in reality impaired in their ability to do so. This is coherent with the lines of argumentation of Morf and Rhodewalt [41], who propose that this narcissistic bias may produce an overestimation of their own level of social functioning and an underestimation of the negative effects of their own behaviors upon others. These bias suggest caution when interpreting the self reports of narcissistic patients who are asked to evaluate their own mental functioning and their interpersonal relationships. We expected that low mindreading ability would also be associated with a low subjective distress, but the data did not confirm this hypothesis.

Although the patients with NPD show low levels of symptomatic distress and low levels of mindreading ability, we failed to find a link between these two elements. Inability to understand one’s own mental states does not adequately explain the fact that narcissistic patients appear unaware of distress arising from rejection and personal failure. Defense mechanisms, biased information processing and cognitive avoidance may more cogently account for the low levels of subjective symptomatic distress experienced by narcissists, rather than impairments of mindreading ability.

Two issues remain unsolved. First, if low subjective distress is not due to mindreading deficits but instead to a motivational component, which motivation would push NPD patients to ignore psychological distress? Maintaining high levels of grandiosity should be the most obvious hypothesis. In other words, the need for social approval and the need to feel superior to others could explain low levels of frustration and depression in these patients. If so, an inverse relationship between grandiosity and subjective distress should be expected. We know that NPD patients show poorer mindreading compared with no PD patients, but we are not aware of the variables involved in mindreading impairment in NPD. One possibility is that mindreading impairment in NPD patients is related to lack of empathy. We aim to investigate these two issues—the relationship between mindreading, grandiosity and lack of empathy—in a second study.

## Study 2

The second study intends to address two questions. First, our aim is to investigate the relationship between grandiosity, mindreading and subjective distress. In the clinical descriptions of narcissism, grandiosity is viewed as the other face of fragility, functioning as a protection against feelings of inferiority, emptiness and dependency, and insulating against experiences of inconsistency and fragmentation of the self. These descriptions of the narcissistic patient would therefore appear to imply that high levels of grandiosity will be associated with low levels of subjective perceptions of distress. Consistently with this hypothesis, we expect to find an inverse relationship between grandiosity and subjective distress.

The second question is: if poor mindreading ability plays no role in the relative absence of subjective distress, what role does it play in narcissistic pathology? To date, we have shown that the mindreading impairment does not appear to be linked to the absence of subjective distress, but we have not tested the hypothesis advanced by several authors that this impairment is linked to a lack of empathy [37]. We expect that low levels of decentering and mindreading ability will stand in the way of competent understanding of other people, and that in consequence these patients will struggle to regulate their interpersonal relationships effectively. We therefore expect to find a significant relationship between low mindreading ability and those criteria for narcissism which impact most severely on patients’ relational capacities: lack of empathy, arrogance and entitlement.

To explore these two hypotheses about the relationship between grandiosity, lack of empathy and mindreading, we described the different role of each single criterion of NPD as it impacts on perceived symptoms severity and mindreading. This analysis was performed on the total sample and without differentiating for any specific diagnosis, in order to investigate the role of each single narcissistic dimension (conceptualized in terms of SCID-II narcissistic criteria) in predicting subjective distress (conceptualized as symptoms severity, GSI) and mindreading (conceptualized as TAS-20 and MAI scores). From a theoretical point of view, we aimed to investigate the specific role of narcissistic dimensions in predicting specific features of mental functioning, independently of a personality disorder diagnosis. In other words, we are here assuming that narcissistic criteria, if present, and even if not corresponding to any categorical diagnosis, can be associated with certain specific forms of dysfunction. We predict that grandiosity will be linked to low levels of subjective distress, and that lack of empathy, independently of NPD diagnosis, will be linked to low levels of mindreading ability.

### Materials and methods

#### Participants

In this second study, we used the whole sample from which we had selected the three patient groups participating in the previous study, without differentiating for any specific diagnosis. A total of 1357 patients took part in the study (605 males, or 44.6% of the sample; 752 females, or 55.4%; mean age 34.21, SD = 10.74, range 18–73). A total of 545 patients had no PD diagnosis, while a total of 812 had at least one PD diagnosis. Patients with neurological disorders, psychotic disorders, and active substance dependence were not included in the sample.

#### Procedure and measures

Measures and procedure corresponded to those of study 1.

### Data analyses

In order to test our explorative hypothesis, we conducted three linear regressions where the dependent variables (TAS-20, GSI and MAI) were regressed on the NPD criteria (entered simultaneously in each equation). Statistical analysis of data was performed using SPSS 20.0.

### Results and discussion

Table 3 shows the correlations matrix among predictors and dependent variables. Table 4 shows the results of the linear regressions on alexithymia, symptoms severity and the global score of MAI. The criterion *grandiosity* was negatively associated with symptoms severity and alexithymia, as hypothesized. Other negative associations resulted between the criteria *sense of entitlement*, *lack of empathy* and *envy*, and the global score of MAI. Positive links were observed between the criteria *fantasies of unlimited success*, *lack of empathy*, *envy* and perceived symptoms severity (GSI), and between *lack of empathy* and alexithymia.

**Table 3:** Correlation matrix of predictors and dependent variables.

	1	2	3	4	5	6	7	8	9	10	11	12	13	14	15	16
1. Grandiosity	1															
2. Unlimited success	.44**	1														
3. Beliefs of being special	.44**	.33**	1													
4. Requests of admiration	.49**	.46**	.38**	1												
5. Sense of entitlement	.40**	.31**	.31**	.38**	1											
6. Interpersonal exploitation	.32**	.38**	.28**	.33**	.45**	1										
7. Lack of empathy	.37**	.32**	.32**	.32**	.36**	.44**	1									
8. Envy	.35**	.36**	.27**	.42**	.29**	.32**	.27**	1								
9. Arrogant attitude	.50**	.32**	.38**	.40**	.34**	.29**	.39**	.29**	1							
10. GSI	-.01	.10**	.01	.03	.06*	.06*	.07**	.13**	.02	1						
11. Alexithymia tot	-.08**	-.00	-.04	-.05	.01	.02	.06*	.01	-.04	.51**	1					
12. Monitoring	-.04	-.05	-.03	-.04	-.09**	-.10**	-.17**	-.09**	-.06*	-.08**	-.18**	1				
13. Differentiation	-.11**	-.14**	-.09**	-.11**	-.20**	-.16**	-.24**	-.16**	-.18**	-.22**	-.19**	-.61**	1			
14. Integration	-.05	-.09**	-.04	-.08**	-.14**	-.13**	-.18**	-.10**	-.11**	-.14**	-.18**	.75**	.73**	1		
15. Decentration	-.13**	-.13**	-.10**	-.10**	-.21**	-.16**	-.22**	-.12**	-.15**	-.09**	-.14**	.61**	.70**	.70**	1	
16. MAI tot	-.09**	-.12**	-.07*	-.09**	-.19**	-.16**	-.23**	-.14**	-.14**	-.15**	-.20**	.85**	.87**	.91**	.87**	1

**p < .01

*p < .05. N = 1207.

**Table 4:** Prediction of Narcissistic criteria in Alexithymia, symptoms severity and MAI.

	Dependent Variables		
Narcissistic criteria	Alexithymia	GSI	MAI tot
**Independent Variables**	B(SE) β	B(SE) β	B(SE) β
1. Grandiosity	-1.49(.52) -.10**	.09(.03) -.11*	.77(.49) .06
2. Unlimited success	.28(.47) .02	.06(.03) .07*	-.35(.44) -.03
3. Beliefs of being special	-.52(.50) -.03	-.02(.03) -.02	.49(.47) .03
4. Requests of admiration	-.44(.43) -.03	-.00(.02) -.00	.36(.41) .03
5. Sense of entitlement	.22(.51) .01	.03(.03) .03	-1.59 (.47) -.11***
6. Interpersonal exploitation	-.01(.55) .00	-.01(.03) -.01	-.35(.52) -.02
7. Lack of empathy	1.72(.50) .11**	.06(.03) .06*	-2.54(.47) -.18***
8. Envy	.31(.43) .02	.10(.02) .12***	-.80(.41) -.06*
9. Arrogant attitude	-.49(.46) -.03	-.01(.03) -.01	-.69(.43) .05

For TAS-20, N = 1357; for GSI, N = 1354; for MAI, N = 1210

***p < .001

**p < .01

*p < .05.

TAS-20 R^2^ = .02.; F (9,1347) = 3.12; p < .001. MAI R^2^ = .08; F (9,1201) = 10.92; p < .001. GSI R^2^ = .03; F (9,1344) = 4.33; p < .001.

Table 5 shows the results of the linear regressions on the four dimensions of MAI. Interestingly, the only positive link that emerged was the one between the first criterion, *grandiosity*, and the dimension *integration*. Significant negative associations were evidenced between *sense of entitlement* and *integration*, *decentration* and *differentiation*. *Lack of empathy* was negatively associated to each dimension of MAI. The criterion *envy* was negatively associated to *monitoring* and *differentiation*. Finally, *arrogant attitude* was negatively associated to the dimension *differentiation*.

**Table 5:** Prediction of Narcissistic criteria in MAI dimensions.

	Dependent Variables			
Narcissistic criteria	Monitoring	Differentiation	Integration	Decentration
**Independent Variables**	B(SE) β	B(SE) β	B(SE) β	B(SE) β
1. Grandiosity	.15 (.14) .04	.23(.13) .06	.31(.14) .08*	.08(.15) .02
2. Unlimited success	.01 (.12) .00	-.15(.12) -.04	-.07(.13) -.02	-.14(.13) -.04
3. Beliefs of being special	.10(.13) .03	.15(.12) .04	.20(.14) .05	.04(.14) .01
4. Requests of admiration	.09(.12) .03	.09(.11) .03	.01(.12) .00	.16(.12) .05
5. Sense of entitlement	-.13(.13) -.03	-.47(.13) -.12**	-.38(.14) -.09*	-.61(.14) -.14**
6. Interpersonal exploitation	-.12(.15) -.03	-.02(.14) -.01	-.14(.15) -.03	-.06(.16) -.01
7. Lack of empathy	-.64(.13) -.16**	-.65(.12) -.17**	-.59(.14) -.14**	-.65(.14) -.15**
8. Envy	-.23(.12) -.06*	-.27(.11) -.08*	-.16(.12) -.04	-.14(.12) -.04
9. Arrogant attitude	-.00(.12) .00	-.29(.11) -.09*	-.21(.13) -.06	-.18(.13) -.05

N = 1210

** p < .001

*p < .05.

*Monitoring* R^2^ = ,04; F(9,1201) = 5,06, p < .001. *Differentiation* R^2^ = ,09; F (9,1201) = 12,89, p < .001. *Integration* R^2^ = ,05; F(9,1201) = 6,98, p < .001. *Decentration* R^2^ = ,08; F (9,1201) = 10,93, p < .001.

We explored the relationships between the various criteria for NPD and symptomatic distress, alexithymia and mindreading. As expected, symptomatic distress stands in inverse relationship to grandiosity. This data is coherent with clinical descriptions which suggest that higher levels of inflated self-esteem protect narcissists from subjective distress [58]. Symptomatic distress also presents a direct relationship with the criterion of *envy*. One possible explanation for this is that heightened envy or heightened perceptions of being the object of envy may indicate that subjects are more aware of difficulties or may be involved in more serious interpersonal conflicts. Considering that previous research studies have highlighted that social impairment may function as a mediator between narcissism and personal distress [15], envy may prove to be linked to symptomatic distress as an emotional marker of social impairment.

*Grandiosity* emerges not only as not linked to poor mindreading ability but as positively associated with all measures of MAI, reaching small but statistical significance with *integration*. This data is partially coherent with Kernberg’s model [14], which asserts that grandiosity provides narcissists with the means for maintaining a relatively integrated self-image, functioning as a protective measure against processes of fragmentation.

A number of NPD criteria emerged in this study as linked to various types of metacognitive impairment, to a greater or lesser degree. The criterion which appears as most tightly linked to low mindreading ability is *lack of empathy*. In line with the definition of empathy in section III of the DSM-5, which also highlights the cognitive aspects of lack of empathy in NPD patients, we found that lack of empathy is linked to poor decentering. It is worth noting that the criterion also presents a significant inverse relationship with other typical self-domain aspects of metacognition such as monitoring and integration [57]. This may indicate that the ability to be empathetic does not only requires an understanding of the minds of others (i.e., *decentering*), but also an ability to read our own minds (i.e., *monitoring and integration*) and a consequent recognition of what we may have in common with others.

The other criteria which are significantly linked to poor mindreading ability are *entitlement*, *envy* and *arrogance*. Taken together, these criteria describe problematic interpersonal attitudes, feelings and behaviours. In consequence, our data may suggest that poor mindreading ability is linked to the interpersonal aspects of narcissistic pathology. We are aware that the effects we found were small, albeit significant. This suggests that poor mindreading plays a role in aspects of narcissistic interpersonal functioning but that other factors should also be taken into consideration.

The results have confirmed our hypotheses, although the limited dimension of the effect does not allow us to draw definitive conclusion. Although based on preliminary and exploratory data, this results may indicate some interesting directions for further investigations. In the first place, these data may serve to enhance understanding of the distinction between high-functioning and low-functioning narcissism. As Calgor and colleagues [9] have proposed, clinical assessment of NPD should include not only the distinction between different subtypes but also distinctions between different levels of severity and personal functioning. If mindreading is linked to the social functioning levels of patients with narcissism, it follows that patients with better mindreading ability may also be capable of devising relational strategies that are overall less counterproductive, leading to fewer interpersonal conflicts, fewer threats to self-esteem and improved social functioning.

Taking into account that social impairment functions as a mediator between narcissism and psychopathology, our data might suggest that mindreading could be one of the mediators between narcissism and social impairment. If this is the case, low levels of mindreading ability could constitute a negative prognostic factor which renders patients more vulnerable to social dysfunction and symptomatic deterioration.

The role played by grandiosity remains an open question. Eminent clinicians have suggested that grandiosity appears to perform a protective role with regard both to the development of symptoms and to mental functioning [14, 16]. Conversely, crises of grandiosity can be accompanied by symptomatic deterioration, fragmentation and diminished integration. Studies of therapeutic process should therefore analyze the relationships between mindreading abilities and grandiosity in order to preclude potentially iatrogenic side effects of therapy.

Some limitations should be acknowledged. First of all, in the first study we chose to focus our analyses on patients diagnosed primarily with NPD, with no other comorbid diagnosis. This decision was taken in order to preclude eventual confusing effects arising from comorbid diagnoses. However, one consequence of this strategy was that the group we studied was smaller than would otherwise have been the case. Another consequence is that the level of severity in this sample was less significant than that usually encountered in clinical practice, where patients generally show a number of comorbidities.

Moreover, this study observed a sample of narcissistic patients with overt grandiosity and it is unclear whether and to what extent our findings may be transferable to the vulnerable phenotype. Further research on the vulnerable phenotype of narcissistic functioning would be required to clarify this. In addition, the exploratory analyses we conducted in the second study produced no insight into the type of causal relationship which may exist between low levels of empathy and low levels of mindreading ability. It is conceivable, for instance, that both low empathy and poor mindreading could depend upon a common motivational factor, bypassing mentalization altogether [39].

Finally, we used only the criteria of the categorical diagnosis. An investigation carried out with dimensional measurements, in line with the alternative model of personality dysfunction proposed in DSM-5, could provide a more exhaustive description of the relationships between dimensions of narcissism and mindreading.

## Supporting information

S1 File Database file. All the variables described in the reported studies are included in the database.
(SAV)
